# CINs of the cytoplasm: dissecting dsRNA signaling in chromosomal instability

**DOI:** 10.1002/1878-0261.70047

**Published:** 2025-05-07

**Authors:** Aglaia Skolariki, Rose L. Jady‐Clark, Eileen E. Parkes

**Affiliations:** ^1^ Department of Oncology University of Oxford UK; ^2^ Nuffield Department of Medicine, Centre for Immuno‐Oncology University of Oxford UK

**Keywords:** chromosomal instability, dsRNA, innate immunity, MAVS, micronuclei, RNA sensing

## Abstract

Chromosomal instability (CIN), a pervasive feature of cancer, promotes tumor evolution and inflammatory signaling, yet its influence on innate immune sensing remains incompletely understood. Ruptured micronuclei, a direct byproduct of CIN arising from missegregated chromosomes, expose out‐of‐context double‐stranded DNA that engages the cGAS‐STING pathway. In their recent study, Sasaki et al. show that micronuclei are also a source of immunogenic double‐stranded RNA (dsRNA), triggering MAVS‐dependent type I interferon responses independently of STING. The authors show that micronuclei undergo aberrant transcription, producing dsRNA from nonexonic, transcriptionally accessible loci, with many species localizing near interferon‐stimulated genes. This work suggests a feedforward loop in which type I interferon signaling reinforces its own activation through transcriptional dysregulation. Using MPS1 inhibition to induce acute CIN, the authors show that MAVS signaling promotes MHC Class I expression and immune cell recruitment. These findings reposition CIN as a dual trigger of innate immunity through cytoplasmic DNA and RNA sensing. Future work should define how these pathways integrate in the context of chronic CIN and evaluate strategies to target DNA and RNA sensing in immune‐edited tumors.

## Graphical abstract

Micronuclei, formed during cell division in chromosomal instability settings, rupture and lead to the accumulation of immunogenic double‐stranded RNA in the cytoplasm, activating MAVS‐dependent interferon signaling and innate antitumor immunity. Aberrant transcription from interferon‐stimulated genes further amplifies this response through a feedforward loop, establishing RNA sensing as a distinct axis of CIN‐induced inflammation.

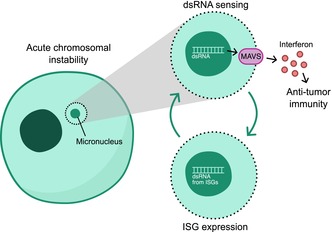

**Abbreviations**
ADAR: adenosine deaminase acting on RNA
cGAS: cyclic GMP‐AMP synthase
CIN: chromosomal instability
CXCL10: C‐X‐C motif chemokine ligand 10
DNMTi: DNA methyltransferase inhibitor
dsDNA: double‐stranded DNA
dsRNA: double‐stranded RNA
ERV: endogenous retrovirus
IFN: interferon
IRF3/7: interferon regulatory factor 3 and 7
ISG: interferon‐stimulated gene
MAVS: mitochondrial antiviral‐signaling protein
MDA5: melanoma differentiation‐associated protein 5
MPS1: monopolar spindle 1
MPS1i: monopolar spindle 1 inhibition
RIG‐I: retinoic acid‐inducible gene I
STAT1: signal transducer and activator of transcription 1
STING: stimulator of interferon genes
TBK1: TANK‐binding kinase 1
ZBP1: Z‐DNA binding protein 1

## 1 Introduction

Chromosomal instability (CIN) is a fundamental driver of tumorigenesis and metastasis, fueling genetic diversity and adaptation under selective pressure [1]. CIN typically arises from mitotic errors that lead to chromosome missegregation and micronuclei formation. These small extranuclear bodies are prone to rupture, exposing double‐stranded DNA (dsDNA) to the cytosol, thus activating the cyclic GMP‐AMP synthase (cGAS)‐stimulator of interferon genes (STING) signaling pathway, priming the expression of interferon (IFN) β and initiating innate antitumor immunity [2, 3]. While the cGAS‐STING axis has often been central to discussions linking CIN to innate immune activation, a growing body of evidence now reveals that the immunogenic consequences of CIN extend beyond cytosolic DNA sensing [4, 5]. In particular, transcriptional dysregulation and RNA processing defects can lead to the cytoplasmic accumulation of immunostimulatory double‐stranded RNA (dsRNA), a potent trigger of antiviral signaling [6].

## 2 The source of cytoplasmic dsRNA in cancer

Transposable elements, notably endogenous retroviruses (ERVs) and long interspersed nuclear elements, are normally epigenetically silenced but can become transcriptionally active under cellular stress and produce RNAs that fold into inverted‐repeat duplexes, most commonly involving Alu elements [7]. They constitute a significant source of self‐derived dsRNA that can be recognized by canonical RNA sensors, such as MDA5 and RIG‐I, which bind dsRNA and oligomerize to activate mitochondrial antiviral‐signaling protein (MAVS), triggering phosphorylation of IRF3/7 and transcription of interferon‐stimulated genes (ISGs), even in the absence of detectable cytosolic DNA or STING activity [8].

Epigenetic derepression of retroelements has also been achieved following treatment with DNA methyltransferase inhibitors (DNMTi), leading to upregulated ERV expression and a ‘viral mimicry’ response via dsRNA accumulation. DNMTi exposure induced an ISG signature in ovarian cancer cell lines via the STING‐independent MDA5‐MAVS axis [9] with similar findings reported in colorectal cancer cell lines [10].

This has significant implications for tumors with epigenetically silenced STING or dysfunctional DNA‐sensing capacity, where MAVS‐mediated RNA sensing may represent a dominant route of immunogenicity. However, whether CIN alone could generate immunostimulatory dsRNA species remains unclear. In their recent publication in *Molecular Cell*, Sasaki et al. [11] demonstrate that chromosome missegregation itself is sufficient to generate dsRNA and drive MAVS‐dependent interferon signaling, reframing micronuclei as transcriptionally active compartments capable of initiating antiviral mimicry.

## 3 Cytoplasmic dsRNA in response to CIN

The authors build on earlier work, establishing pulsed inhibition of the spindle assembly checkpoint MPS1 (MPS1i) as a potent driver of chromosome missegregation and micronuclei formation [12]. Following mitotic errors and re‐entry into G1, the micronuclei undergo aberrant transcription, including readthrough of noncoding regions and intron retention, leading to cytoplasmic dsRNA release. Using dsRNA immunoprecipitation with the J2 antibody followed by dsRNA‐sequencing, the authors traced the genomic origins of dsRNA to nonexonic regions, including repetitive elements and loci with high chromatin accessibility [11]. Intriguingly, many of the IFN‐dependent dsRNA peaks mapped to loci adjacent to ISGs, a pattern also observed following exogenous IFN‐β treatment. This suggests that type I IFN signaling reinforces its own activation via chromatin remodeling and aberrant transcription at ISG loci, establishing a feedforward loop that sustains immunogenic dsRNA production.

To functionally validate whether dsRNA accumulation following chromosome missegregation engages the RNA‐sensing machinery, the authors performed transcriptomic analyses in MAVS‐, STING‐, and MAVS/STING‐deficient non‐small cell lung cancer cell lines [11]. MAVS‐dependent gene clusters were enriched for type I interferon response genes, but the MPS1i‐induced cytokine response was markedly attenuated in MAVS‐depleted cells, with the most profound suppression observed in double knockouts. While STING‐deficient cells retained cytokine output, suggesting STING independence, MAVS loss dampened phosphorylation of TBK1 and STAT1, and IFN‐β and CXCL10 secretion. Importantly, dsRNA accumulation was preserved across all genotypes, confirming MAVS as a downstream effector rather than a regulator of dsRNA biogenesis.

The authors further examined the regulation of the dsRNA sensing axis by depleting the RNA‐editing enzyme ADAR, which suppresses dsRNA immunogenicity via adenosine‐to‐inosine conversion [11]. ADAR depletion significantly enhanced immune activation following MPS1i; however, these effects were abolished by co‐depletion of MAVS, confirming that the response in ADAR‐deficient cells was MAVS‐mediated.

Finally, MAVS‐dependent signaling upregulated MHC class I expression and enhanced natural killer cell chemotaxis using a 3D microfluidic system that mirrored *in vivo* immune engagement [11]. Syngeneic 129S2/SvPasCrl mouse models bearing cGAS‐deficient 393P lung tumors were studied, revealing that MAVS‐deficient tumors were resistant to pulsed MPS1i therapy. In contrast, MAVS reconstitution restored IFN‐β and CXCL10 secretion, CD8^+^ T‐cell infiltration, and granzyme B expression. These effects were lost in immunodeficient mice, confirming that MAVS‐driven responses rely on host immunity.

## 4 Cytoplasmic dsRNA in context in CIN‐high cancer

The evidence provided by Sasaki et al. indicates that immunogenic dsRNA is generated as a consequence of CIN, thereby broadening our understanding of how innate immunity is shaped in this context and offering a potential pathway for therapeutic exploitation beyond STING agonists. Yet, the study models acute CIN using short‐term MPS1i, whereas many epithelial tumors sustain chronic CIN and undergo adaptive immune editing and selective pressure over time, including rewiring of the cGAS‐STING pathway to promote tumor growth [13, 14]. Whether MAVS‐mediated RNA sensing is similarly rewired under chronic stress remains unknown.

Additionally, the observed ADAR dependence following MPS1i reveals a potential therapeutic vulnerability [11]. This aligns with prior work showing that cancer cells with chronic STING‐driven ISG signatures are primed for dsRNA sensing and selectively sensitive to ADAR loss [15]. Deletion or inhibition of ADAR in this setting could, therefore, amplify immune signaling, potentially converting CIN from a tolerance‐inducing stressor to a trigger of immunogenic cell death. However, the heterogeneity of ADAR dependence across cell lines indicates a need for biomarkers that can stratify tumors based on their immune editing capacity. Furthermore, combination treatments should be carefully considered, as MPS1 inhibitors are not without toxicity, and the precise window for inducing immunogenic CIN without provoking widespread cell death remains to be defined [16].

**Fig. 1:**
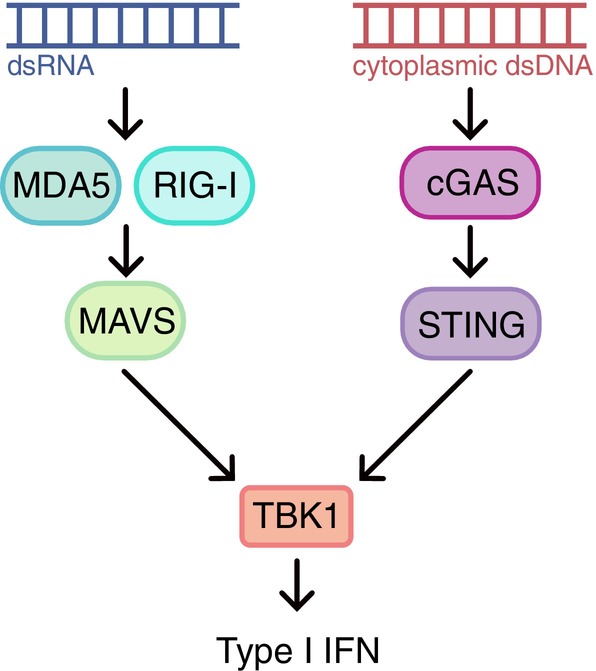
Cytoplasmic double‐stranded RNA and DNA signal through innate immune sensors, MDA5/RIG‐I and cGAS, respectively, to activate parallel MAVS‐ and STING‐dependent signaling cascades. These distinct pathways converge at the kinase TBK1, which coordinates the downstream induction of type I interferon responses.

Finally, recent evidence suggests that an expanding repertoire of long noncoding RNAs can activate innate immune pathways [17]. ZBP1, an innate immune sensor, signals through MAVS and is induced by STING, pointing to potential crosstalk between DNA‐ and RNA‐sensing pathways. Altogether, these insights converge on a broader framework in which cytosolic nucleic acids trigger TBK1‐dependent signaling cascades (Fig. 1). Defining how these pathways interact and integrate across CIN states will be critical for leveraging nucleic acid sensing in cancer immunotherapy.

## Conflict of interest

AS is an employee of the University of Oxford and has received funding or other support for research work from Nucana and the Hellenic Society of Medical Oncology. EEP has served on advisory boards and received fees from companies, including Boehringer Ingelheim, Curadev, InhaTarget, and AkamisBio. She is an employee of the University of Oxford, which has received funding or other support for research work from AstraZeneca and STIpe Therapeutics.

## Author contributions

AS and EEP contributed to the conceptualization, discussion, and analysis of Sasaki et al. AS contributed to the writing—original draft. RLJ‐C contributed to the visualization. EEP contributed to the supervision. AS, RLJ‐C, and EEP contributed to the writing—review and editing.
